# Epicardial adipose tissue in obesity-related heart failure with preserved ejection fraction and atrial fibrillation: mechanisms, imaging, and therapeutic modulation

**DOI:** 10.3389/fendo.2026.1976969

**Published:** 2026-10-09

**Authors:** Qi Zhu, Tiance Xu, Chen Yang, Lili Tan

**Affiliations:** 1 Department of Emergency, Central Hospital Affiliated to Shenyang Medical College, Shenyang, China; 2 Department of Neurology, Central Hospital Affiliated to Shenyang Medical College, Shenyang, China; 3 Department of Endocrinology, Central Hospital Affiliated to Shenyang Medical College, Shenyang, China; 4 Department of Cardiology, Central Hospital Affiliated to Shenyang Medical College, Shenyang, China

**Keywords:** atrial fibrillation, cardiac imaging, epicardial adipose tissue, heart failure with preserved ejection fraction, obesity, therapeutic modulation

## Abstract

Obesity-related heart failure with preserved ejection fraction (HFpEF) and atrial fibrillation (AF) often coexist, but the contribution of epicardial adipose tissue (EAT) to their overlap remains uncertain. EAT is anatomically contiguous with the myocardium and can undergo inflammatory, profibrotic and metabolic remodeling in obesity. In HFpEF, greater EAT burden is associated with higher filling pressures and findings compatible with external cardiac restraint; in AF, regional peri-atrial fat is associated with abnormal conduction, fibrosis and ablation outcomes. Human EAT experiments demonstrate local profibrotic signaling, while autonomic interventions involving epicardial fat pads provide procedural evidence that does not isolate adipocyte effects. Recent invasive observations in patients with AF also link CT-defined EAT to atrial pressures and obesity-related HFpEF. Exercise, weight loss and metabolic therapies can change measured cardiac fat, although the relevant compartments and responses vary. CT attenuation is not a direct or uniformly directional measure of EAT inflammation. EAT is therefore a measurable phenotype at the HFpEF–AF interface, with stronger local experimental evidence in AF than in HFpEF. Whether changing EAT itself contributes to clinical benefit beyond systemic weight loss or other treatment effects requires longitudinal studies in patients with both conditions.

## 1 Introduction

Obesity identifies a distinct clinical phenotype of heart failure with preserved ejection fraction (HFpEF), characterized by greater volume expansion, higher filling pressures and impaired exercise reserve (1). Epicardial adipose tissue (EAT) has been associated with invasive hemodynamic abnormalities and functional limitation in HFpEF (2, 3), raising the question of whether cardiac fat provides information beyond overall adiposity.

Atrial fibrillation (AF) frequently coexists with HFpEF and can further compromise atrial and ventricular function. Experimental sustained obesity produces atrial enlargement, conduction abnormalities and greater AF susceptibility (4). In human mapping studies, electrical changes have been more pronounced near epicardial fat depots (5). Such observations motivate investigation of local cardiac adiposity alongside systemic obesity-related risk factors.

EAT lies within the visceral pericardium and directly contacts the heart. Human tissue studies have described an inflammatory and adipocytokine profile that differs from subcutaneous adipose tissue (6, 7). The proximity of EAT to ventricular, coronary and atrial structures provides a potential route for local interactions, although proximity alone does not demonstrate a disease-causing effect.

Epidemiological studies have linked the depot reported as pericardial fat to AF and atrial conduction indices (8, 9), as well as AF severity and ablation outcomes (10). In experimental human-tissue systems, EAT-derived factors have induced atrial fibrosis or activated atrial fibroblasts (11, 12). These different levels of evidence make the HFpEF-AF overlap a pertinent setting in which to examine EAT, provided that the measured fat compartment and the type of evidence are distinguished.

This review considers the biology of EAT in obesity, the clinical and experimental evidence in HFpEF, AF and their overlap, methods for measuring defined cardiac fat compartments, and treatment-associated changes. It distinguishes associations from local mechanistic experiments and asks what additional evidence is needed to establish whether EAT modification contributes to clinical benefit.

## 2 EAT remodeling in obesity

### 2.1 Anatomical definition and physiological functions

Epicardial adipose tissue (EAT) lies between the myocardial surface and the visceral pericardium, with prominent deposits around the coronary vessels, atrioventricular grooves and atria. It provides local fatty-acid storage and supply, expresses thermogenic markers such as UCP1 and secretes adipokines including adiponectin (13–15). Paracardial fat lies outside the parietal pericardium. The label pericardial fat is protocol-dependent: some studies restrict it to the intrapericardial space, whereas others include both inner and outer depots. We therefore report the original investigators’ anatomical definition rather than treating these labels as synonyms.

### 2.2 Obesity-associated EAT remodeling

Obesity-related EAT expansion is accompanied in some human disease cohorts by adipocyte and secretory changes. In coronary-disease specimens, EAT had an inflammatory and adipocytokine profile distinct from subcutaneous fat (6, 7); EAT-derived adipokines also affected monocytes and endothelial cells experimentally (16). The experimental observations support local inflammatory signaling but have not established an EAT-specific cause of HFpEF or AF.

EAT phenotype varies by cardiometabolic setting and cellular composition, including its immune-cell populations (17). Coronary perivascular fat attenuation can reflect adjacent arterial inflammation (18), but that perivascular measure is not interchangeable with global or peri-atrial EAT attenuation. Volume and CT attenuation therefore describe different aspects of cardiac fat; their biological interpretation depends on the sampled compartment.

## 3 Clinical associations and experimental evidence linking EAT, HFpEF and AF

### 3.1 HFpEF: hemodynamic associations and proposed ventricular pathways

Clinical HFpEF studies associate greater EAT burden with higher invasive filling pressures and poorer exercise hemodynamics (2, 3). Pugliese et al. also related echocardiographic EAT thickness to hemodynamic and metabolic features and prognosis in heart failure (19), while Crum et al. reported findings compatible with greater pericardial restraint in HFpEF (20). Jin et al. found associations between EAT thickness and left atrial and ventricular function (21). These studies describe a cardiac-adiposity phenotype but cannot separate local fat effects from body size, cardiac geometry or systemic metabolic factors.

Inflammatory and mechanical pathways offer different possible explanations. Human EAT can release inflammatory and profibrotic mediators, and EAT-derived products can affect vascular cells in experimental systems (6, 11, 16); the initiation of coronary microvascular dysfunction by EAT in patients with HFpEF has not been demonstrated. Because EAT accumulates within the pericardial space, it might also contribute to external cardiac restraint, particularly when diastolic reserve is limited. The relative contributions of local signaling and pericardial mechanics remain unresolved.

An EAT-specific lipotoxic pathway remains a hypothesis. Experimental work on obesity-associated myocardial lipid overload links excess fatty-acid uptake and accumulation of lipid intermediates to organelle stress and cardiomyocyte dysfunction (22). A review focused on EAT describes possible local lipid-mediated injury but does not demonstrate directional EAT-to-myocardium lipid flux in human HFpEF (23). Myocardial fat and interstitial fibrosis have also been examined in relation to contractile function (24), and EAT imaging in relation to HFpEF diagnosis and prognosis (25). None of these findings establishes EAT-derived fatty-acid transfer as the cause of mitochondrial dysfunction, abnormal calcium handling or impaired relaxation in patients with HFpEF.

The atrial component of HFpEF merits separate attention: increased EAT has been associated with impaired left atrial mechanical function in patients with preserved or mildly reduced ejection fraction (26). Whether this association persists independently of ventricular filling pressures, AF burden and systemic adiposity is an open question, considered in the overlap population below.

### 3.2 AF: regional fat, atrial substrate and local experimental evidence

The anatomical region measured matters for AF associations. In 1,946 Framingham participants, greater CT-defined pericardial fat was associated with longer P-wave duration in both sexes: per 1-SD increase in fat, adjusted beta coefficients were +2.3 ms in women and +1.2 ms in men; P-wave terminal force was also higher in sex-stratified models (9). A separate Framingham analysis related pericardial fat to prevalent AF (8). Other cohorts associated the depot termed pericardial fat with AF type, severity or ablation outcomes (10, 27), whereas Batal et al. quantified left atrial epicardial adiposity (28). CMR has separately associated EAT with left atrial volume and fibrosis in patients with AF (29). The reported associations are consistent with adverse atrial conduction rather than improved conduction, although the measured fat compartments are not necessarily identical.

Histological studies provide a distinct line of evidence. Haemers et al. described fibrotic remodeling of subepicardial adipose tissue in human and sheep atria with AF (30). Ishii et al. linked fibrotic remodeling of EAT adjacent to the left atrial appendage with persistent AF and corresponding CT attenuation changes (31). These tissue findings suggest that fat composition and spatial relationships may be relevant alongside fat volume.

Human EAT-conditioned media induced atrial myocardial fibrosis in experiments implicating Activin A (11); atrial EAT from patients with AF has also been shown to secrete myeloperoxidase and activate atrial fibroblasts (12). Greater EAT Activin A expression has been associated with postoperative AF in cardiac-surgery patients (32). The ex vivo experiments establish that EAT-derived factors can act on atrial tissue, but their contribution to AF progression in patients has not been quantified.

Electrophysiological evidence connects local fat distribution with atrial substrate. Nalliah et al. studied cardiac-surgery patients without established AF using CT, high-density epicardial mapping of the anterior right atrium, histology and co-culture; local EAT accumulation was associated with conduction slowing, electrogram fractionation, fibrosis and altered connexin distribution (33). Obesity models, atrial-myocyte co-culture and mapping studies in patients with AF provide complementary, but not interchangeable, evidence (5, 34–36). The spatial and experimental data support local electrical effects without establishing that the same mechanism initiates AF or HFpEF in patients.

Epicardial fat pads also contain autonomic ganglionated plexi. Singhal et al. assessed CT-defined regional left atrial epicardial fat and atrial electrogram dominant frequency in 32 patients undergoing AF ablation (37). During sinus rhythm, they defined high-dominant-frequency electrogram sites (DF >70 Hz) as AF nests and found regional associations with epicardial fat. These refer to electrogram spectral properties, not sites receiving high-frequency electrical stimulation; the study did not demonstrate that fat cells rather than embedded neural structures caused the observed association.

### 3.3 HFpEF–AF overlap: clinical interaction and shared adiposity

Elevated ventricular filling pressures in HFpEF can impair left atrial function, whereas AF removes coordinated atrial contraction and may further restrict filling and exercise reserve (38–40). These reciprocal physiological effects make it important to distinguish the contribution of cardiac fat from consequences of AF and ventricular disease. The association of EAT with left atrial mechanical dysfunction in preserved or mildly reduced ejection fraction provides an additional link (26).

The HFpEF–AF overlap has now been studied directly. Ariyaratnam et al. enrolled 120 symptomatic AF patients with preserved ejection fraction undergoing ablation; the study used an invasively measured mean left atrial pressure criterion to define an HFpEF phenotype (41). The 44 obese participants had higher mean left and right atrial pressures and a higher prevalence of HFpEF than the 76 nonobese participants. CT-defined EAT volume and total cardiac volume were greater in the obese group and each correlated with mean right atrial pressure, whereas global left atrial voltage and reservoir strain did not significantly differ by obesity status. These observations link the directly measured cardiac-fat compartment to the hemodynamic phenotype in an AF cohort and are compatible with greater external cardiac restraint. The fat–pressure correlation was not a mediation analysis, however, and cannot isolate EAT from total cardiac volume, systemic adiposity or AF-related hemodynamic changes.

Further studies in patients with both conditions should measure AF burden, atrial mechanics, filling pressures, body composition and anatomically defined cardiac fat in the same participants. The STEP-HFpEF analysis additionally shows that patients with AF and obesity-related HFpEF can experience improvements in HF symptoms and functional measures with semaglutide (88), but it did not establish whether peri-atrial EAT changed or AF burden improved. Such combined designs may clarify whether regional peri-atrial fat and intrapericardial burden contribute different information after accounting for systemic adiposity and cardiac geometry.

**Figure 1** summarizes reported HFpEF and AF associations with obesity-related EAT remodeling and indicates that the proposed local links remain incompletely established.

**Figure 1:**
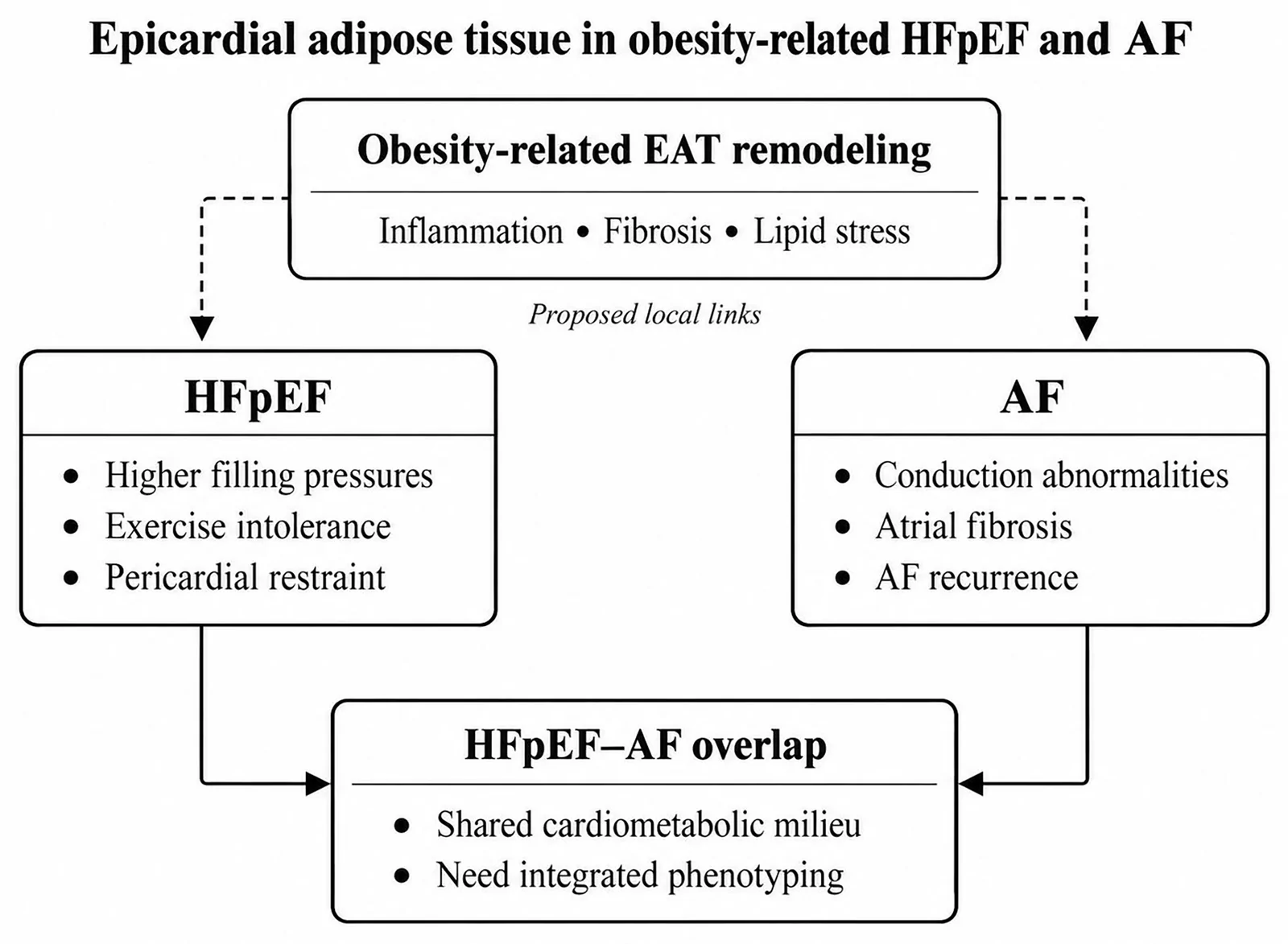
Proposed epicardial adipose tissue (EAT) links to obesity-related heart failure with preserved ejection fraction (HFpEF) and atrial fibrillation (AF). The dashed arrows indicate candidate local relationships rather than established direct causality. The ventricular and atrial findings are clinical associations, while inflammatory, fibrotic and lipid-stress pathways have varying degrees of experimental support. The lower convergence denotes the HFpEF–AF overlap phenotype, not a demonstrated causal sequence between diseases.

## 4 Imaging assessment and phenotyping of EAT

### 4.1 Echocardiography: accessible screening but limited phenotyping

Transthoracic echocardiography provides an accessible measure of EAT thickness, typically over the right ventricular free wall. The approach described by Iacobellis et al. (42) and subsequent work on cardiometabolic associations (43) allow cardiac adiposity to be assessed alongside standard ventricular and atrial measurements without dedicated cross-sectional imaging.

Its practicality supports serial assessment, including in exercise studies (44, 45). However, right ventricular thickness cannot represent total EAT volume or peri-left atrial distribution, and no validated echocardiographic EAT threshold determines HFpEF or AF treatment. Acoustic-window quality and the respiratory or cardiac phase may affect measurement reproducibility.

For questions about regional fat, atrial substrate or tissue attenuation, echocardiography should be complemented by a clearly defined CT or CMR measurement rather than treated as an interchangeable volume estimate.

### 4.2 CT-based EAT volume and attenuation

CT can quantify total and regional cardiac fat volume and attenuation in Hounsfield units, provided the segmentation boundaries and acquisition protocol are defined. Total EAT, posterior left atrial fat and peri-pulmonary vein fat may answer different questions. Studies of ablation outcomes have measured different pericardial or epicardial depots (10, 46), underscoring the need to report the actual region rather than use EAT as a generic label. A meta-analysis also examined associations between volumetric EAT and cardiac structure and function (47).

CT fat attenuation captures aspects of tissue composition but is not a histological assay. Lipid content, edema, fibrosis, vascularity and scanner protocol may affect the measured value (48–50). Coronary perivascular attenuation reflects a different compartment from total or peri-atrial EAT; treatment-related attenuation changes are considered in Section 5.1.

Regional AF studies have related peri-left atrial EAT radiodensity, posterior fat attenuation, enhancement and attenuation dispersion to atrial electrophysiology or ablation outcomes (51–54), with additional reports on peri-atrial fat quality, EAT volume or attenuation (55–57). A 2025 meta-analysis reported higher left atrial EAT attenuation in patients with AF recurrence (58). However, a separate 2025 meta-analysis of 16 studies (4,175 patients) found no overall association between EAT attenuation and recurrence, whereas the subset with consistent or corrected tube voltage showed a modest association between higher attenuation and lower recurrence risk (59). Differences in CT protocol, anatomic region and outcome definition prevent attenuation from being assigned a single clinical meaning.

### 4.3 CMR and integrated cardiac phenotyping

CMR can assess cardiac fat alongside chamber volumes, function and myocardial tissue characteristics. In the SUMMIT substudy, EAT and fat outside the parietal pericardium were measured separately and then summed as paracardiac adipose tissue (60). At 52 weeks, the between-group reduction concerned the combined compartment, driven predominantly by the outer pericardial component; isolated EAT did not differ significantly between tirzepatide and placebo. The combined change must therefore not be presented as demonstrated EAT reduction.

Regional atrial fat can also be assessed using CMR. Ballatore et al. associated epicardial atrial fat indexed to left atrial volume with recurrence after cryoballoon ablation (61). CMR permits such fat measures to be considered alongside left atrial function and ventricular remodeling, whereas CT is commonly available from pre-ablation anatomical imaging.

CMR fat quantification varies with sequence, segmentation and normalization. Studies should distinguish epicardial fat from combined fat compartments and report whether values are indexed to body surface area, atrial volume or another denominator. Reproducible compartment definitions are more important than selecting a single preferred modality.

### 4.4 Radiomics, artificial intelligence and inflammatory EAT phenotyping

Automated CT segmentation can reduce manual workload and provide volume and attenuation estimates; CMR segmentation has also been explored (62, 63). Reproducibility still depends on acquisition, anatomical boundaries and external validation.

Radiomic approaches extract regional texture and intensity features beyond average fat volume or attenuation. Peri-left atrial EAT radiomics and dispersion have been associated with AF recurrence after ablation (54, 64). These associations do not establish that an individual radiomic feature directly measures inflammation or fibrosis. Work on coronary perivascular fat provides an adjacent, but anatomically distinct, imaging precedent (65).

### 4.5 Measurement boundaries and research applications

Modality selection should follow the research question: echocardiography for accessible right ventricular thickness, CT for regional volume and attenuation, and CMR for combined fat and cardiac functional assessment. These are complementary measurements, not steps in a sequential imaging algorithm.

For cross-study comparability, reports should define epicardial, pericardial and paracardial boundaries, specify CT acquisition and fat-segmentation settings, state any indexing method and document reproducibility. In SUMMIT, separate EAT and outer pericardial measurements were available, but the significant treatment effect concerned their combined paracardiac volume (60); this outcome must not be renamed isolated EAT.

## 5 Therapeutic modulation of EAT

### 5.1 Interpreting changes in EAT burden and phenotype

Changes in EAT amount and attenuation should be interpreted separately. In the STOP randomized CT substudy, semaglutide reduced intrapericardial EAT volume by approximately 9% and increased its mean attenuation by approximately 5% over 12 months in participants with type 2 diabetes and coronary artery disease (66). The attenuation increase means that the Hounsfield-unit value became less negative; it does not by itself indicate either resolution or worsening of inflammation. Some studies of peri-left atrial fat have associated higher attenuation with AF recurrence (52, 58), but other studies have reported differing directions. A meta-analysis of 16 studies found no overall association with recurrence; in the subset with consistent or corrected tube voltage, higher attenuation was modestly associated with lower recurrence risk (59). STOP measured global intrapericardial EAT on noncontrast CT, whereas AF studies used different regional definitions and acquisition protocols. Lipid content, adipocyte size, edema, fibrosis and vascularity may all affect attenuation, and the available scans cannot distinguish these mechanisms. Thus, a lower EAT volume and a less negative CT density should not be combined into an unvalidated composite marker of healthier tissue.

Most serial intervention studies were conducted in obesity, type 2 diabetes or broader heart-failure cohorts, not in patients with both HFpEF and AF. They show that an anatomically defined fat compartment may change during treatment; they do not establish that the fat change mediates improvement in symptoms, filling pressures or arrhythmia outcomes. **Figure 2** separates intervention, imaging and clinical endpoints accordingly. Representative serial imaging studies are summarized in **Table 1**, and tissue or cell experiments are presented separately in **Table 2**.

**Figure 2:**
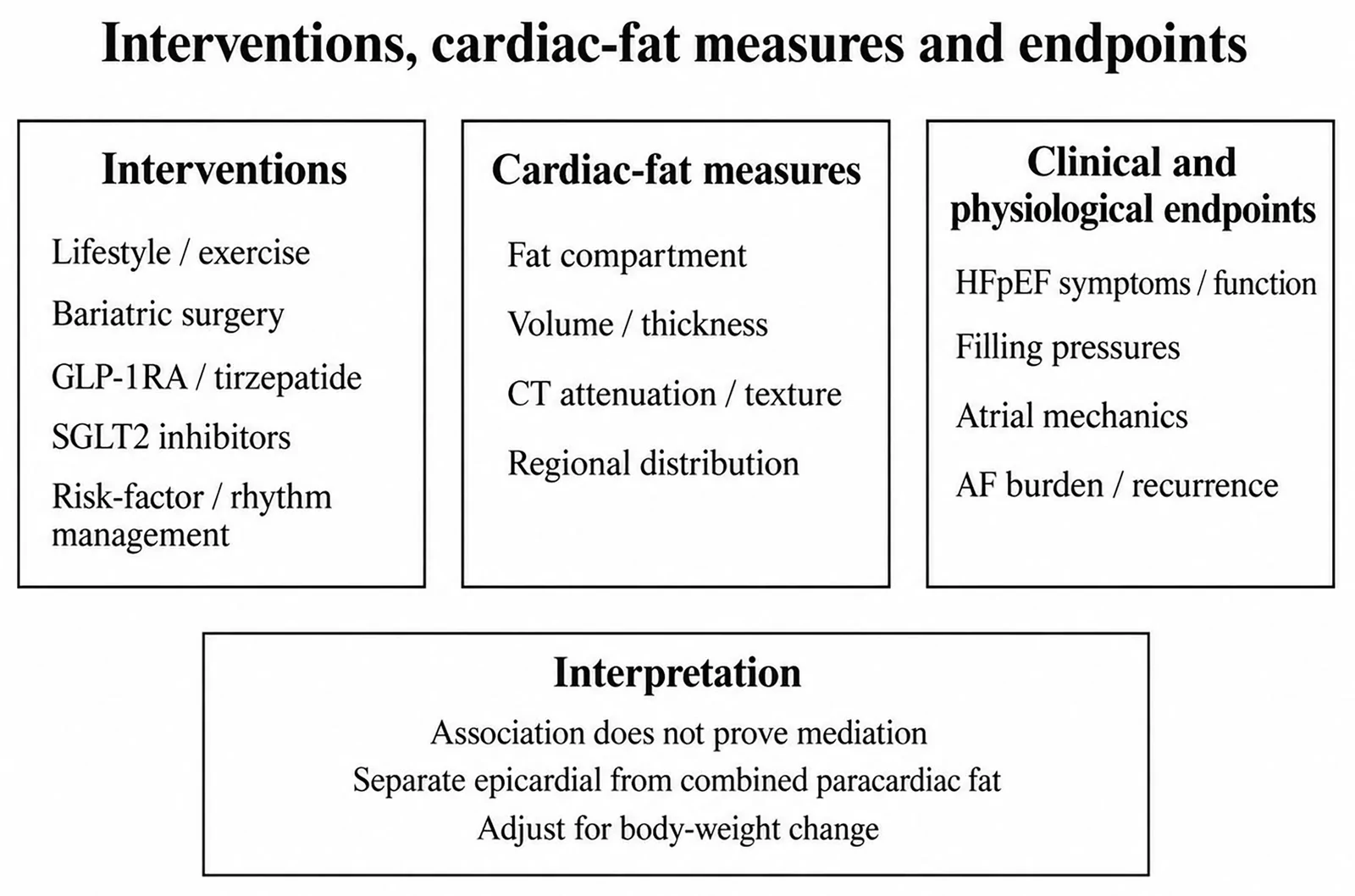
Interventions, cardiac-fat measurements and clinical or physiological endpoints for studies of obesity-related HFpEF and AF. The columns are deliberately unconnected: changes in cardiac adipose tissue have not been established as mediators of clinical benefit. The measured fat compartment should be defined, and CT attenuation should not be interpreted as a direct inflammatory readout. Associations with outcomes require consideration of overall body-weight change.

**Table 1:** Representative serial human imaging studies of cardiac adipose tissue.

Study/population	n; follow-up	Intervention/design	Measured depot; method	Reported cardiac-fat change	Weight-loss adjustment/mediation
Christensen et al. (67); abdominal obesity	50 randomized; 39 analyzed; 12 wk	Endurance or resistance vs control; RCT secondary analysis	Epicardial fat mass; MRI	Within-arm: -32% endurance; -24% resistance. Relative to control: -56% and -48%.	Weight-independent mediation not assessed
Gaborit et al. (71); severe obesity	23 paired; 6 mo	Bariatric surgery; prospective cohort	Epicardial fat volume; MRI	137 ± 37 to 98 ± 25 mL; -27 ± 11%	Percent EAT change not correlated with percent BMI change (r=0.14, P = 0.53); no mediation test
Chin et al. (73); obesity without known cardiac disease	142 completed; 12 mo	Bariatric surgery; prospective cohort	Epicardial fat thickness; echocardiography	4.9 ± 1.5 to 3.4 ± 1.1 mm (-31%)	BMI-adjusted associations with function; not evidence of mediation by EAT reduction
van Eyk et al. (82); South Asian T2D	47 randomized; 26 wk	Liraglutide vs placebo; RCT	Epicardial and paracardial volumes, separately; MRI	Neither depot showed a significant between-group difference	No significant cardiac-fat effect detected
Rasmussen et al. (81); T2D	102 randomized; 26 wk	Liraglutide vs placebo; RCT secondary analysis	Cardiac adipose volume as defined in study; CT	Between-group difference: -11.4 mL (95% CI -19.4 to -3.3)	Difference not significant after BMI-change adjustment (P = 0.46)
STOP/Manubolu et al. (66); T2D and CAD	115 eligible; 90 analyzed; 12 mo	Semaglutide vs placebo; RCT substudy	Intrapericardial EAT volume and density; noncontrast CT	~9% lower EAT volume; ~5% higher density vs placebo in multivariable models	Weight-independent EAT mediation not tested
Sato et al. (93); T2D and CAD	35 analyzed; 6 mo	Dapagliflozin (18) vs conventional care (17); *ad hoc* analysis of earlier trial (98)	EAT volume; CT	EAT volume change: -15.2 ± 12.8 vs +3.0 ± 11.9 cm³ (dapagliflozin vs conventional care; P = 0.01)	Weight change differed between arms (−3.0 vs +0.3 kg); EAT-specific mediation not tested
EMPACEF/Gaborit et al. (103); T2D	56 randomized; 12 wk	Empagliflozin vs placebo; RCT	Epicardial fat volume; MRI	No significant between-group difference (P = 0.88)	No detectable EAT effect
SUMMIT/Kramer et al. (60); obesity-related HFpEF	175 baseline; 106 paired; 52 wk	Tirzepatide vs placebo; RCT CMR substudy	Combined paracardiac fat (EAT + external pericardial); CMR	Combined fat: -45 mL placebo-corrected (95% CI -69 to -22); isolated EAT: no significant difference	No selective EAT change demonstrated; weight-independent mediation untested

Sample sizes and effect estimates refer to the specified analysis populations and measured fat compartments.

AF, atrial fibrillation; BMI, body mass index; CAD, coronary artery disease; CMR, cardiac magnetic resonance; CT, computed tomography; EAT, epicardial adipose tissue; HFpEF, heart failure with preserved ejection fraction; MRI, magnetic resonance imaging; RCT, randomized controlled trial; T2D, type 2 diabetes. Within-group and placebo-corrected changes are not interchangeable. Adjustment for baseline BMI or BMI change does not itself establish EAT-specific mediation. When a weight-independent effect or mediation test was not reported, this is stated rather than inferred.

**Table 2:** Human EAT tissue-endpoint and ex vivo studies.

Study	Study design/human material	Exposure/readout	What can be concluded
Díaz-Rodríguez et al. (105)	EAT samples from 52 cardiac-surgery patients; explant treatment (n=21) and adipocyte assays	Dapagliflozin; insulin resistance, chemokines and adipocyte differentiation	Direct ex vivo exposure; no patient-level serial EAT measurement
Iacobellis et al. (83)	38 patients with T2D and CAD randomized to pre-CABG liraglutide or placebo; regional EAT and subcutaneous fat sampled at surgery	4–12 weeks of *in vivo* liraglutide or placebo; coronary/left atrial EAT microRNAs and intraoperative glycemia	Single-time-point tissue endpoint after randomized clinical exposure; not an ex vivo drug-exposure trial and no serial EAT imaging
Basdas et al. (84)	Human EAT-derived stromal/adipogenesis systems and hiPSC-derived atrial cardiomyocytes	Semaglutide; inflammatory adipogenesis and paracrine readouts	Ex vivo/cellular paracrine observations; no patient-level serial EAT measurement or demonstrated clinical mediation

A randomized preoperative tissue-sampling study is distinguished from direct exposure of explants or cell systems; none measured serial patient-level EAT reduction.

To assess an EAT-specific effect, future trials should measure cardiac fat and clinical outcomes at prespecified time points and account for concurrent changes in body weight, other adipose depots and treatment-related cardiovascular effects.

### 5.2 Lifestyle intervention, exercise and surgical weight loss

Lifestyle modification targets the upstream metabolic stress that drives EAT expansion. Kim et al. showed that 12 weeks of aerobic exercise reduced echocardiographic EAT thickness in obese middle-aged men, and the reduction in EAT paralleled improvement in abdominal visceral adiposity (44). Bairapareddy et al. also reported reduced EAT thickness after aerobic exercise in overweight adults (45). In a randomized exercise trial, Christensen et al. found that both endurance and resistance training reduced cardiac adipose tissue, with modality-specific effects on different fat compartments (67). A pilot randomized trial in type 2 diabetes subsequently showed that caloric restriction, with or without additional physical activity, reduced epicardial fat, with a larger reduction in the combined-intervention group (68). In obese older patients with HFpEF, caloric restriction and aerobic exercise training improved peak oxygen consumption and quality of life, supporting lifestyle intervention as a clinically relevant background strategy even when EAT was not the primary endpoint (69).

Bariatric surgery provides stronger and more durable weight loss and has been associated with substantial EAT reduction. Willens et al. reported a significant decrease in echocardiographic EAT thickness after bariatric surgery-induced weight loss (70). Gaborit et al. used magnetic resonance imaging and spectroscopy to show that bariatric surgery reduced epicardial fat volume, whereas myocardial triglyceride content did not significantly change, indicating that cardiac ectopic fat depots do not respond identically to weight loss (71). In a serial CMR study, Henry et al. further showed that changes in epicardial and visceral adipose tissue after bariatric surgery were accompanied by changes in cardiac geometry and markers of pericardial restraint (72). More recently, Chin et al. reported a 31% reduction in echocardiographic EAT thickness one year after bariatric surgery; EAT reduction was associated with improved systolic and diastolic indices after covariate adjustment, but this observational analysis did not establish EAT-mediated improvement independent of change in weight (73). The Swedish Obese Subjects cohort associated bariatric surgery with lower incident AF risk, without establishing mediation through EAT (74).

### 5.3 Glucagon-like peptide-1 receptor agonists and dual incretin therapy

Incretin-based therapies may modify cardiac fat alongside body weight and glycemic control. Early EAT-focused studies examined liraglutide, exenatide, semaglutide and dulaglutide with different imaging methods (75–80). Serial studies report heterogeneous findings: the LIRAFLAME randomized substudy found a CT-defined cardiac-fat difference of -11.4 mL with liraglutide at 26 weeks, but the result was no longer significant after adjustment for change in body mass index (81). A separate 26-week placebo-controlled MRI trial in 47 South Asian patients with type 2 diabetes found no significant between-group change in either epicardial or paracardial fat despite weight loss (82). In STOP, 90 participants had analyzable serial CT scans, with the volume and attenuation changes discussed in Section 5.1 (66). The available serial studies do not establish a weight-independent EAT-mediated clinical effect. A randomized preoperative liraglutide study with a single-time-point human EAT microRNA endpoint and a separate ex vivo semaglutide paracrine study are summarized with their distinct designs in **Table 2** (83, 84).

Disease-specific evidence in obesity-related HFpEF comes from the STEP-HFpEF program. In STEP-HFpEF, once-weekly semaglutide 2.4 mg improved heart failure-related symptoms, physical limitations and exercise function and produced greater weight loss than placebo in patients with HFpEF and obesity (85). In STEP-HFpEF DM, similar benefits were observed in patients with obesity-related HFpEF and type 2 diabetes (86). A pooled analysis of STEP-HFpEF and STEP-HFpEF DM confirmed consistent improvements in symptoms, body weight and functional outcomes across key subgroups (87). These trials did not primarily test EAT as the mediator, so the relationship between their clinical benefits and changes in cardiac adipose tissue remains unresolved.

In a STEP-HFpEF Program analysis, Verma et al. reported that AF was present in nearly one-half of patients with obesity-related HFpEF and was associated with features of more advanced heart failure; semaglutide improved heart failure-related symptoms, physical limitations and exercise function and reduced weight, C-reactive protein and N-terminal pro-B-type natriuretic peptide in patients with and without AF (88). Because AF was common and semaglutide remained beneficial across AF strata, metabolic treatment remains relevant after AF has become part of the HFpEF phenotype. Whether these benefits are accompanied by changes in peri-atrial EAT, atrial mechanics or AF burden has not yet been established.

Tirzepatide reduced the composite risk of cardiovascular death or worsening heart failure and improved health status in the SUMMIT trial (89). Its CMR substudy included 106 patients with evaluable paired scans at 52 weeks and found a 45-mL placebo-corrected reduction in combined paracardiac adipose tissue (epicardial plus external pericardial fat); the isolated EAT component did not differ significantly between groups (60). Thus, SUMMIT establishes change in a combined cardiac fat compartment, not selective EAT reduction or mediation of HFpEF benefit by EAT. Weight loss and changes in cardiac structure are potential co-occurring treatment effects.

### 5.4 SGLT2 inhibitors and cardiometabolic modulation

SGLT2 inhibitors are central to the treatment of HFpEF, but their relationship with EAT is less consistent than that reported for incretin-based therapy. In EMPEROR-Preserved, empagliflozin reduced the combined risk of cardiovascular death or hospitalization for heart failure in patients with HFpEF (90). In DELIVER, dapagliflozin reduced worsening heart failure or cardiovascular death in patients with mildly reduced or preserved ejection fraction (91). The 2026 IDEAL-AF randomized trial found greater improvement in echocardiographic diastolic function with SGLT2 inhibition than with dipeptidyl peptidase-4 inhibition in patients with AF and diabetes (92). IDEAL-AF did not measure EAT, so this clinical effect cannot be attributed to cardiac adipose remodeling.

Serial cardiac-fat studies with SGLT2 inhibitors have been inconsistent. Some diabetes cohorts reported reductions in EAT thickness or CT-defined volume (93–101). The 2020 Sato analysis (93) re-examined participants from the previously reported dapagliflozin trial (98), so these publications are not independent cohorts. By contrast, an empagliflozin-versus-sitagliptin randomized study found no significant between-group cardiac-fat reduction (102), and the 56-participant EMPACEF randomized trial found no significant MRI EAT-volume change after 12 weeks (103). Hsu et al. similarly found no significant six-month change in CMR-defined pericardial fat (104). Díaz-Rodríguez et al. obtained EAT from 52 cardiac-surgery patients and investigated dapagliflozin in tissue and adipocyte experiments, including explant assays; they did not measure EAT change in treated patients (105). That study therefore informs tissue biology, not clinical fat reduction, and is presented only in **Table 2**.

Evidence in broader cardiovascular populations is also mixed. In nondiabetic patients with heart failure with reduced ejection fraction, the prespecified EMPA-TROPISM secondary analysis found a significant CMR EAT reduction with empagliflozin compared with placebo (106). The SIMPLE substudy reported a reduction in cardiac adipose tissue volume assessed using rubidium-82 positron emission tomography/computed tomography (107), while a more recent pooled analysis of randomized dapagliflozin trials reported EAT reduction in patients with heart failure and type 2 diabetes (108). In the 2026 Malaysian EpiCAD prospective observational cohort of patients with type 2 diabetes and coronary artery disease, EAT thickness decreased by 29% in the SGLT2-inhibitor group and increased by 13% in the non-SGLT2-inhibitor group over 6 months; the nonrandomized design limits causal interpretation (109). A 2026 meta-analysis of 11 serial imaging studies involving 284 patients found an overall reduction in EAT volume or thickness with SGLT2 inhibition, but the pooled studies were small and heterogeneous in design and imaging modality (110). SGLT2 inhibition can improve HFpEF outcomes even when EAT change is unknown or modest, so serial EAT imaging is better treated as a mechanistic endpoint than as a prerequisite for therapy.

### 5.5 Local autonomic interventions and rhythm-focused risk management

Local autonomic interventions have been evaluated in patients undergoing cardiac surgery, a population distinct from chronic HFpEF-AF. In a randomized study of 60 patients with paroxysmal AF undergoing coronary artery bypass grafting (CABG), botulinum toxin injection into four posterior epicardial fat pads was associated with a lower incidence of atrial tachyarrhythmias from day 30 through 36 months compared with placebo (23.3% vs 50%; hazard ratio 0.36, 95% CI 0.14–0.88) (111). In the pCAD-POAF randomized trial of 430 patients undergoing isolated CABG, ligament-of-Marshall division together with Waterston-groove fat-pad resection reduced newly occurring AF during the first 6 postoperative days (18.1% vs 31.6%; risk ratio 0.57, 95% CI 0.41–0.81) in participants without preoperative AF (112). The first study followed patients with pre-existing paroxysmal AF, while the second addressed new-onset postoperative AF. Both interventions target neural structures within or adjacent to fat pads; they do not isolate the effect of reducing EAT adipocytes, and their outcomes cannot be extrapolated directly to chronic HFpEF-associated AF.

EAT-derived Activin A and myeloperoxidase can activate profibrotic pathways in tissue or cell experiments (11, 12), but no selective clinical blockade of these adipose-derived signals has demonstrated improved HFpEF or AF outcomes. These tissue findings are considered mechanistic observations, not evidence that a tested anti-inflammatory treatment reverses EAT burden or disease. Tissue and cell-based evidence is summarized separately in **Table 2**.

Weight reduction can also alter AF disease trajectory. In the observational REVERSE-AF analysis of 355 patients with BMI at least 27 kg/m2 offered weight and risk-factor management, 88% of those with baseline persistent AF who achieved at least 10% weight loss reverted to paroxysmal or no AF, compared with 26% in the group with less than 3% weight loss (113). Progression from paroxysmal to persistent AF was 3% versus 41% across the same weight-loss groups. These are subgroup-specific changes in AF classification within a nonrandomized cohort, not percentages of the entire 355-patient sample or proof of mediation by EAT. The earlier observational ARREST-AF and LEGACY studies likewise associated sustained risk-factor management with AF outcomes (114, 115), and the randomized ARREST-AF trial tested comprehensive risk-factor management alongside ablation (116). None established serial peri-atrial EAT change as a mediator.

## 6 Discussion

The evidence for candidate local EAT contributions is more mechanistically developed in AF than in HFpEF. AF research combines regional imaging, atrial mapping, histology and experiments using human EAT-derived factors (11, 12, 30, 33), while studies in HFpEF predominantly associate cardiac fat burden with filling pressures, exercise physiology and external restraint (2, 3, 20). In a recent 120-patient AF cohort, invasively measured atrial pressures and CT-defined EAT were assessed together (41), but the cross-sectional design does not establish whether EAT contributes to, or reflects, the hemodynamic phenotype. In particular, the ventricular effects of local EAT-derived signaling in human HFpEF remain less directly established than experimental atrial effects.

Interventions also require separate interpretation. Weight management is associated with changes in AF trajectory (113), and clinical trials have demonstrated benefits of semaglutide or tirzepatide in obesity-related HFpEF (85–89). Serial fat imaging, however, has not shown that these benefits are mediated by EAT. The SUMMIT CMR substudy reported a change in combined paracardiac fat, not a significant isolated EAT reduction (60). Botulinum-toxin fat-pad injection and combined fat-pad resection/denervation were studied in cardiac-surgery patients (111, 112); effects on local autonomic pathways cannot be ascribed to selective removal of EAT adipocytes or extrapolated directly to chronic HFpEF-associated AF.

The apparently divergent CT attenuation findings illustrate a measurement problem rather than an established biological contradiction. In STOP, global intrapericardial EAT volume fell as attenuation increased (66), whereas regional AF studies reported heterogeneous associations between attenuation and recurrence (52–59). Attenuation depends on the sampled depot, lipid and nonlipid tissue components, and CT acquisition; it cannot serve as a direct inflammatory readout without tissue or other independent validation. For similar reasons, echocardiographic thickness, CT volume and CMR-defined combined fat cannot be pooled as though they measured a single exposure. No validated EAT threshold currently determines treatment choice in either HFpEF or AF.

A prospective study of patients with both HFpEF and AF would provide a more direct test of the shared-substrate hypothesis. It should obtain reproducible regional and total fat measurements, systemic adiposity, cardiac geometry, atrial mechanics, filling pressures, AF burden and clinical endpoints at prespecified intervals. Randomized interventions with serial measurements could then establish whether fat change precedes clinical improvement and test mediation after accounting for weight loss and other treatment effects. Until such evidence is available, EAT imaging is a research phenotype rather than an independent target for therapeutic decisions.

## 7 Conclusion

EAT lies close to the ventricular myocardium and atria and is associated with features of both obesity-related HFpEF and AF. Clinical HFpEF evidence remains mainly observational; AF studies add regional mapping and local tissue experiments, but do not establish that EAT is the common causal driver of the two conditions. Changes in cardiac fat during metabolic treatment and local autonomic procedures do not yet demonstrate an EAT-specific clinical benefit. Longitudinal studies with consistent anatomical definitions and direct assessment of the HFpEF–AF overlap are needed to determine whether EAT modification adds benefit beyond systemic cardiometabolic treatment.
